# Enuresis Nocturna in children with asthma: prevalence and associated risk factors

**DOI:** 10.1186/s13052-016-0266-3

**Published:** 2016-06-10

**Authors:** Emin Ozkaya, Seren Calıs Aydın, Mebrure Yazıcı, Rusen Dundaröz

**Affiliations:** Department of Pediatrics, Division of Pediatric Allergy and Immunology, Bezmialem Vakif University Medical Faculty, Adnan Menderes Bulvari Vatan Caddesi, 34093 Fatih/Istanbul, Turkey; Department of Pediatrics, Giresun University Medical Faculty, Giresun, Turkey; Department of Pediatrics, Bezmialem Vakif University Medical Faculty, Adnan Menderes Bulvari Vatan Caddesi, 34093 Fatih/Istanbul, Turkey

**Keywords:** Asthma, Enuresis, Children, Risk factors, Autonomic dysfunction, Pollen allergy, Eosinophilia

## Abstract

**Background:**

Enuresis Nocturna (EN) is a common disorders in childhood. Although many different underlying pathophysiological mechanisms have been proposed to explain EN, its etiology is multifactorial. Some reports demonstrate that there is an association between EN and allergic diseases.

To study (1) the prevalence of EN in children with asthma, (2) to determine the possible risk factors for EN in asthmatic children.

**Methods:**

Five hundreds and six children aged 6–14 years-old diagnosed with asthma and 380 age-matched non-asthmatic controls were enrolled into this cross-sectional case–control study. We studied an allergy panel that included skin prick tests with (8 inhalant allergens), total IgE, and blood eosinophil count for both groups. Semi-structured interviews were conducted with the parents of children presenting EN. Factors associated with EN in children with asthma were analyzed using a logistic regression model.

**Results:**

The prevalence of EN was significantly higher in children with asthma as compared to the controls: 132 (26 %), 43 (11.5 %) respectively (*p* = 0.001). Emergency visits frequency, and family history of enuresis were higher in the asthmatic children with EN than in asthmatic children without EN. According to the logistic regression analysis, positive pollen sensitization (*p* = 0.027, OR = 1.94), allergic rhinitis (*p* = 0.032, OR = 2.36), and high eosinophil count (*p* = 0.004, OR = 1.40) were independent risk factors for EN in children with asthma.

**Conclusion:**

This study showed that the prevalence of EN in children with asthma was higher than in same age controls. Sensitization to pollens, allergic rhinitis and high blood eosinophil count associate to the EN in children with asthma.

## Background

Asthma is the most common chronic childhood disease [1]. The cause of asthma is not yet completely understood, and there is no consensus about its etiology. Various factors have been implicated in the pathogenesis of childhood asthma [2], such as combined contribution of genetic predisposition environmental insults. A great deal of interest has recently been raised concerning the relationship between psychosocial factors and asthma morbidity [3–5].

Enuresis Nocturna (EN), common in childhood. Several theories have been proposed to explain the ıts pathogenesis, such as developmental delay, increased urine output secondary to decrease in nocturnal antidiuretic hormone, detrusor hyperactivity, psycho-behavioral problems, sleep disorders [6–9]. Although many different underlying pathophysiological mechanisms have been proposed to explain EN, its etiology is multifactorial.

Some reports demonstrate an association between EN and allergic diseases [10, 11]. Avoiding food and inhaled allergens resulted in alleviation of the symptoms of asthma and EN [12]. Also some studies showed that total immunoglobulin E (IgE) level was higher than normal in 60 % of the asthmatic children with EN, and that levels of eosinophilic cationic protein (ECP) are significantly higher than in controls [10, 13]. It has also been showed that there is general or local bladder hypersensitivity in asthmatic children that associated with increased parasympathetic tone in both of diseases [14, 15].

The objective of this study was to evaluate the prevalence of EN in children with asthma and to determine risk factors in cases presenting both diseases occur concomitantly.

## Methods

### Participants

We conducted a cross-sectional case -control study, 650 children diagnosed with stable mild-to-moderate atopic asthma from the authors’ Bezmialem Vakif University Hospital Pediatric Allergy-Pulmonology outpatient clinic were enrolled consecutively between April 2011 and June 2013. The diagnosis and severity of asthma were defined according to Global Initiative for Asthma (GINA) [16] guidelines. Children were defined as asthmatic according to the following criteria:

- recurrent episodes of at least one symptoms of asthma, including cough, wheezing, breathlessness, and chest tightness;
- an improvement of at least 12 % in baseline forced expiratory volume in 1 s (FEV1) after bronchodilator use.

A study questionnaire requesting demographic data and family history of atopy was obtained to each patient. Children with asthma which showed also allergic rhinitis symptoms or were prescribed rhinitis treatment previously or intermittently were included. We did not use polysomnography for diagnosis obstructive sleep apnea syndrome (OSAS), that may associated with EN in asthmatic children who have got allergic rhinitis symptoms due to ethical and financial problems. Informed consent was given by the family of the patients. Asthmatic children presenting lower urinary track system (LUTS) symptoms, structural urologic disease or urinary tract infections after an examination by Pediatric Nephrologists were excluded from the study. After exclusions the total number of the asthmatic children participating was 506.

The control group consisted of age matched 380 children (6–14 years), chosen from those who were periodically attending pediatric welfare clinics of the same hospital for regular checkups. Control patients were evaluated with regard to chronic and/or severe infections, autoimmune disorders, familial history of atopy, eosinophil counts and total IgE level. Children were included in the control group if they had no personal familial history of atopy, no signs of atopic disorder, and if they were negative for skin prick test.

### Blood samples

Venous blood samples were taken from each patient Whole blood analysis was done and total serum IgE was measured. Serum samples for in vitro total IgE were obtained from children after 8 h of fasting, were than centrifuged for 10 min at 1200 rpm and stored at –20 °C. Total serum IgE (IU/mL) was measured on an Immulite 2000 automated analyzer (DPC) using the chemiluminescent enzyme immunoassay method. Eosinophil counts were determined from venous blood samples using a Coulter Counter for leukocyte measurements (Beckman Coulter, Inc.).

### Skin prick tests

Skin prick tests for major allergens (grass mixture, tree mixture, cereal weeds, mould mixture, cat, dog, cockroach, and house dust mites (HDM) - Dermatophagoides pteronyssinus, Dermatophagides farinea) were performed on the forearm of each patient (Allergopharma®, Germany). Histamine dihydrochloride (10 mg/ml) and saline were used as positive and negative controls, respectively. The test was considered positive if the average diameter of the weal was at least 3 mm bigger than the negative control.

### Enuresis Nocturna (EN) survey

Enuresis was defined as bed-wetting during sleep in children ≥ five years of age, according to the International Children’s Continence Society (ICCS) [17, 18]. EN can be isolated (monosymptomatic EN) or associated with daytime lower urinary tract (LUT) symptoms (Nonmonosymptomatic EN) and is subdivided into primary and secondary forms. We did not obtained detailed urological anamnesis that may be related to hyperactive bladder (non-monosymtomatic enuresis). For this reason, we only focused in this study prevalence of primary monosymptomatic EN in children with asthma rather than nonmonosymptomatic EN or secondary EN.

A semi-structured interview was designed by the authors. During the interview, which was held in the out-patient clinic, One member of the study team (SC) directly asked the questions about EN to the patients and/or caregivers. Patients were asked if they ever wet the bed at night more than once a week for at least three months after the age of five [17]. Paternal schooling, family income, daily life problems (school attainment, occupational or academic failure), family problems (conflicts and/or quarrels with family members), disease-related variables (frequency of hospitalization for asthma attacks in the previous year) and some demographic characteristics were also investigated. All children with a history of EN were consulted by the pediatric nephrology department of the same hospital for the further etiological evaluation. After a detailed urologic evaluation, 144 children diagnosed with structural, or infectious problems or having daytime were excluded from the study.

### Statistical analysis

Data were analyzed by using the SPSS version 16.5 (SPSS Inc., Chicago, IL, USA). Descriptive statistics (including mean and median for continuous variables, and frequencies for ordinal and nominal variables) were used to describe the data. *χ*^2^ test was used for the comparison of normally distributed categorical variables. Normally distributed parametric variables were compared between groups by using Student’s *t* test. A bivariate analysis was initially studied in order to examine differences between patients with or without EN using Pearson’s *χ*^2^ test. Following bivariate analysis, logistic regression analysis was performed to estimate the prevalence of EN according to each potential confounder. Data were expressed as odds ratio (OR) and 95 % confidence interval (CI). The *p* value <0.05 was considered to be statistically significant.

## Results

Table 1. summarizes the some clinical and demographic variables in study and control groups. Age and sex were similar between the groups. The prevalence of EN was 26 % (*n* = 132) in patients with asthma, while it was 11.5 % (*n* = 43) in control group (*p* = 0.001). A family history of enuresis was more prevalant in children with asthma plus EN than in those presenting isolated asthma (*p* = 0.034, Table 2).

**Table 1:** Some clinical and demographic characteristics of asthmatic children and controls

	Patients *N* = 506	Controls *N* = 380	*p* value
Age (mean) yrs,	8.40 ± 2.60	8.8 ± 1.90	0.69
Sex (male/*n*,%)	290(57 %)	198(52 %)	0.75
EN (*n*, %)	132(26 %)	43(11.5 %)	0.001
Eosinophils/mm3 (mean ± SD)	386 102 ± 33)	168(56 ± 27)	0.025
Family history of Atopy (*n*,%)	204(40 %)	35(17 %)	0.005

*EN*, enuresis nocturna

Values are given as mean ± standard deviation

**Table 2:** Study variables in children with asthma according to the presence Nocturnal Enuresis

	Asthmatic children with NE *N* = 132	Asthmatic children without NE *N* = 374	*p* value
Age (mean) yrs,	7.3 ± 2.1	5.7 ± 1.5	0.038
Sex (male/*n*,%)	75(57 %)	198(52 %)	0.750
Family history of enuresis	55(41 %)	101(26 %)	0.034
Serum IgE levels (IU/mL)	86.7 ± 25	70.6 ± 81	0.583
Eosinophil/mm^3^ (mean ± SD)	386(102 ± 33)	168(56 ± 27)	0.025
Family history of Atopy (n,%)	204(40)	35(17)	0.034
Emergency visit (No, mean ± SD)	4.2(1.2)	2.8(0.7)	0.032
Positive allergic sensitization (n, %)	89(67)	150(40)	0.016
Pollen sensitization (n, %)	53(40)	62(16)	0.024

*NE*, nocturnal enuresis

Values are given as mean ± standard deviation

Positive skin test ratios among the asthmatic children with and without EN was 67 % and 40 %, respectively. Sensitization against all tested allergens was higher among the asthmatic children with EN than those without (*p* < 0.001, Table 2). Specifically for grass mixture, tree mixture and cereal mixture sensitization ratio in asthmatic children with EN was significantly higher than for those without EN (*p* < 0.001). Sensitization to other common aeroallergens (HDM, mold, cat, dog, and cockroach) was not significantly different between asthmatic children with and those without EN (*p* > 0.05, Table 2).

Among asthmatic children with enuresis, education level of the father and family history of atopy was statistically higher than in controls (*p* < 0,05, Table 2). The frequency of emergency visits during the last year was higher in children with asthma plus EN when compared with those without (*p* =0.035). There was no significant difference between enuretic and non-enuretic asthmatic children in terms of total IgE (*p* = 0.058, p > 0.05), but eosinophils count in asthmatic children with enuresis was statistically higher than in those without (p < 0.05, Table 2).

After adjusting for age and gender a logistic regression analysis was performed. As shown in Table 3, positive sensitization to pollens in skin prick test [OR = 1.94, 95 % CI, 1.47–4.48; *p* = 0.027] high eosinophils count [OR = 1.40, 95 % CI, 1.63–3.27; *p* = 0.004], and additional allergic rhinitis diagnosis [OR = 2.36, 95 % CI, 1.86–4.94; p = 0.032] were independent risk factors for EN in children with asthma.

**Table 3:** Multivariate logistic regression analysis of sensitization pattern, total IgE, eosinophil count, and additional allergic rhinitis in asthmatic children with NE (*N* = 132)

	OR	95 % CI	*p* ^*^
Positive sensitization to pollens	1.94^*^	[1.47–4.48]	
Eosinophil count	1.40^*^	[1.63–3.27]	
Plus Allergic rhinitis	2.36^*^	[1.86–4.94]	

*NE*, nocturnal enuresis

^*^ *p* <0.05

## Discussion

Worldwide prevalence of EN ranges between 5 and 10 % at ten years of age [19, 20]. The reported prevalence of EN in healthy Turkey primary school-aged children ranges between 9 and 16 % [21, 22]. Our cross-sectional data show an overall prevalence of EN of 26 % in asthmatic children. This rate is statistically higher than EN incidence in healthy controls and our country average. Recent and past literature on the prevalence of EN in children with allergic diseases is very limited [10, 13]. Also there is no published study primarily focused on prevalence of EN- associated risk factors in asthmatic children. This is the first paper to investigate the prevalence of EN in children with asthma.

Following allergic diseases, enuresis is the second most common chronic disorder in childhood [18], and leads to considerable psycho-behavioral problems and stress in affected children and their caregivers [23]. Its pathogenetic factors are nocturnal polyuria, detrusor over-activity and reduced arousability. Psychological and psychiatric aspects, genetics and obstipation play an additional role in the etiology [24]. Most of the studies of enuretic children do not show any relationship between anatomical defects and NE [25, 26]. Therefore, it is though that EN may have a functional basis. Current evidence suggests that EN has multifactorial etiology which may underlie the different pathophysiologic mechanisms [18].

It has been suggested that some urinary disorders may be associated with allergy [13, 27]. EN was found to be associated with a reported history of asthma and allergic sensitization to common allergens [27]. Respiratory problems may determine or worsen enuresis. Jesus et al, reported in their cohort study a high incidence (54.3 %) of respiratory/ENT problems (asthma and adenoid hypertrophy) in children with complicated bladder dysfunction [28]. A metaanalysis with 3550 children with sleeping breath disorders showed that one-third had enuresis and that adenotonsilectomy provided relief of the urinary symptoms in half of the patients [29]. In contrast to these studies, Siegel et al [30], reported that there was no relationship between allergic disease and EN in school-age children.

The reasons that relate allergic disease and EN are uncertain. Both diseases show similarities, such as familial background, multifactorial and genetic base. One of the pathophysiologic mechanisms in the etiopathogenesis of EN is increased detrusor muscle activity due to excessive autonomic activation during sleep in children associated with autonomic nervous system dysregulation [31]. Researchers suggested that the parasympathetic nervous system hyperactivity may be a cause of vesical hyperactivity in enuretic children. Similarly, Emin et al [15], found a significant relationship between parasympathetic nervous system hyperactivity and disease severity in children with asthma. Our study results also related a higher prevalence of EN in children with uncontrolled asthma (as suggested by frequent emergency visits), as compared to well controlled asthmatic patients. Bed-wetting in EN and cough in asthma are both night-time symptom [32]. Brockmann et al., reported that excessive autonomic activation during sleep in children is a risk factor for EN [33]. Therefore, some autonomic nervous system dysregulation mechanism may partly explain both nocturnal cough in asthma and EN. We did not obtaine a detailed urological anamnesis that might have detected to hyperactive bladder symptoms (non-monosymtomatic enuresis) in our asthmatic cohort, and cannot claim directly the possibility of autonomic imbalance in asthmatic children with EN.

Kaplan et al [34] reported that there were no differences in allergen sensitization to major food allergen between enuretic children and age-matched controls. In contrast to this study, Mungan et al [10] reported that there may be a relationship between EN and some food allergens. We did not research food allergens in our cohort due to restricted study budget.

It has been suggested that the bladder may be as sensitive as other end-organs like nose, lung, and skin for allergy [27]. After the inhaled allergens reaches the allergen-specific antibodies on mast cells or basophiles, mediators such as histamine, prostaglandins and other cytokines are released and promote smooth muscle contraction and mucus secretion. Smooth muscle contraction is well-known effect of all kind of inhalant allergens in the allergic reaction [35]. It was reported that allergens may decrease functional bladder capacity, cause bladder inflammation and smooth muscle contraction [11]. Zaleski et al, reported that EN is associated significantly with hay fever, urticaria and drug allergy [13]. Also, some studies showed that there is increased incidence of nocturnal detrusor contraction in EN [36, 37]. Our study provides evidence for an increased prevalence for EN in children with asthma with any pollen allergy. This independent associations remained when adjusting for other risk factors including age, gender, family history of atopy, and total IgE level suggesting that concurrent pollen sensitization is an independent risk factor for increased EN prevalence in children with asthma.

Nasal obstruction is one of the most prominent symptom in allergic rhinitis [38]. Aydil et al reported that obstructive upper airway problems are significant risk factors for EN in children [39]. Some studies report on the association between allergic rhinitis (AR) and sleep-disordered breathing (SDB) in children [40]. A systematic rewiev showed enuresis in 1/3 of SDB children, in a cohort of 3350 cases [29]. In our study, allergic rhinitis in children with asthma was associated with increased risk for EN in asthmatic children. Obstructive upper airway problems and systemic allergic inflammation in children with both allergic rhinitis than asthma may be associated risks of EN in children. We could not obtaine further laboratory tests like polysomnography for diagnosis obstructive sleep apnea syndrome (OSAS) that may associated with EN in asthmatic children who have got allergic rhinitis symptoms due to ethical and financial problems. This is as a limitation of the our study.

Eosinophilic cystitis (EC), is a rare inflammatory disorder of the bladder of uncertain etiology Despite many case reports in both the adult and pediatric population, its etiology remains unclear. It is thought to be incited by an IgE mediated attraction of eosinophils to the bladder wall with subsequent mast cell degranulation. A history of previous systemic allergic conditions is frequently elicited from patients with EC. Presenting symptoms of EC are most frequently irritative in nature and mimic those of urinary tract infection: enuresis, dysuria, frequency, urgency, and hematuria [41]. Spark et al., reported that 27 (50 %) of the 54 patients with EC demonstrated either a significant eosinophilia or eosinophiluria [42]. In our study, a high eosinophils count was an as independent risk factors for EN in children with asthma. We did not perform further laboratory tests (eosinophiluria, cystoscopy, biopsy) for diagnosis of EC in our study population and cannot affirm that EN in children with asthma is associated with EC.

## Conclusion

Asthma and enuresis frequently co-exist and enuresis is more frequent in asthmatic children than in controls. Asthmatic children with EN display increased blood eosinophil counts, pollen sensitization and allergic rhinitis when compared to children without enuresis. Pediatricians should be aware of the association between asthma and EN so that effective treatment can be offered to families.
